# RXLR and CRN Effectors from the Sunflower Downy Mildew Pathogen *Plasmopara halstedii* Induce Hypersensitive-Like Responses in Resistant Sunflower Lines

**DOI:** 10.3389/fpls.2016.01887

**Published:** 2016-12-19

**Authors:** Quentin Gascuel, Luis Buendia, Yann Pecrix, Nicolas Blanchet, Stéphane Muños, Felicity Vear, Laurence Godiard

**Affiliations:** ^1^Laboratoire des Interactions Plantes Microorganismes, INRA, CNRS, Université de ToulouseCastanet Tolosan, France; ^2^INRA, UMR1095Clermont-Ferrand, France

**Keywords:** *Plasmopara halstedii*, downy mildew, resistant sunflower, oomycete effectors, hypersensitive response, *Agrobacterium*-mediated transient expression, subcellular localization

## Abstract

*Plasmopara halstedii* is an obligate biotrophic oomycete causing downy mildew disease on sunflower, *Helianthus annuus*, an economically important oil crop. Severe symptoms of the disease (e.g., plant dwarfism, leaf bleaching, sporulation and production of infertile flower) strongly impair seed yield. *Pl* resistance genes conferring resistance to specific *P. halstedii* pathotypes were located on sunflower genetic map but yet not cloned. They are present in cultivated lines to protect them against downy mildew disease. Among the 16 different *P. halstedii* pathotypes recorded in France, pathotype 710 is frequently found, and therefore continuously controlled in sunflower by different *Pl* genes. High-throughput sequencing of cDNA from *P. halstedii* led us to identify potential effectors with the characteristic RXLR or CRN motifs described in other oomycetes. Expression of six *P. halstedii* putative effectors, five RXLR and one CRN, was analyzed by qRT-PCR in pathogen spores and in the pathogen infecting sunflower leaves and selected for functional analyses. We developed a new method for transient expression in sunflower plant leaves and showed for the first time subcellular localization of *P. halstedii* effectors fused to a fluorescent protein in sunflower leaf cells. Overexpression of the CRN and of 3 RXLR effectors induced hypersensitive-like cell death reactions in some sunflower near-isogenic lines resistant to pathotype 710 and not in susceptible corresponding lines, suggesting they could be involved in *Pl* loci-mediated resistances.

## Introduction

Sunflower (*Helianthus annuus* L.) is the fourth most important oil crop in world trade after oil palm, soybean and rapeseed, with an annual production of 12.6 million tons of oil. Sunflower produces a healthy oil rich in unsaturated fatty acids as well as a high vitamin E content. Total sunflower production has doubled worldwide over the past 20 years reaching 45 million tons of seeds in 2013 (FAOstat).

Downy mildew caused by *Plasmopara halstedii* is one of the major diseases affecting sunflower yield (Gascuel et al., 2015). This pathogen has been reported in most of the sunflower seed producing countries. Yield loss has been estimated in France to be around 3.5% of commercial seed production but can be up to 100% in contaminated fields. In addition cultivation of sunflower has to be abandoned in highly contaminated fields. *P. halstedii* is an obligate biotrophic oomycete able to infect annual species of the *Helianthus* genus, including *H. argophyllus, H. debilis*, and *H. petiolaris*, and wild and cultivated varieties of sunflower *H. annuus*. *P. halstedii* belongs to the Peronosporales, the most devastating group of plant pathogenic oomycetes which includes the hemibiotroph genus *Phytophthora* causing late blight diseases and a large group of obligate biotrophs causing downy mildews, such as *P. viticola* on grapevine, *Bremia lactucae* on lettuce, and *Hyaloperonospora arabidopsidis* on *Arabidopsis thaliana* (Michelmore and Wong, 2008; Thines and Kamoun, 2010; Gessler et al., 2011). Since 1992, this pathogen has been subject to quarantine regulation in the European Union. Thirty six pathotypes of *P. halstedii*, often known as races, have so far been identified worldwide. They are defined by an international nomenclature system, based on differential virulence profiles on a set of sunflower inbred lines containing different resistance loci called *Pl* (Tourvieille de Labrouhe et al., 2012; Gascuel et al., 2015).

To avoid downy mildew attacks, modern sunflower cultivars carry one or more dominant *Pl* resistance genes. More than 20 *Pl* loci (*Pl1* to *Pl21, Pl_Arg_, Pl_PMI3_*), conferring resistance to at least one pathotype of *P. halstedii*, have been described and 13 of them mapped in sunflower in six main clusters localized on five different linkage groups (LG) (LG 1, 2, 4, 8, and 13), but none has been cloned so far (see for review, Gascuel et al., 2015; Qi et al., 2015, 2016). Resistance to pathotype 710 of *P. halstedii*, a widely spread pathotype in France, is observed with different *Pl* loci (Gascuel et al., 2015). *Pl5, Pl6* and *Pl7* are three *Pl* loci conferring resistance to pathotype 710, for which near-isogenic introgressed lines are available. *Pl5* originated from *H. tuberosus* (a perennial species) (Vranceanu et al., 1981), *Pl6* was derived from wild non-cultivated *H. annuus* and *Pl7* from *H. praecox* Englem and Gray (Miller and Gulya, 1991). *Pl6* and *Pl7* are localized in a large genomic region of LG 8 called cluster *Pl6/Pl7*, close to, but genetically different from the cluster *Pl1/Pl2*, which does not confer resistance to pathotype 710 (Vear et al., 1997). The *Pl6* region is rich in Toll/interleukin-1 receptor- nucleotide binding site- leucine-rich repeat (TIR-NBS-LRR) resistance gene analogs (RGA) (Bouzidi et al., 2002). *Pl5* is localized on LG 13 in another large resistance cluster known as *Pl5/Pl8* and rich in RGA presenting coiled-coil (CC)-NBS-LRR domains (Radwan et al., 2003, 2004, 2008). *Pl21*, another *Pl* locus that does not confer resistance to pathotype 710, is also closely linked (8 cM) to the *Pl5/Pl8* cluster (Vincourt et al., 2012).

Plant-pathogenic oomycetes, either obligate biotrophs or hemibiotrophs, rely for their developmental cycle on pathogenicity factors, called effectors, that modify the metabolism of host plants to their benefit and thus enable pathogenicity (Bozkurt et al., 2012). Repertoires of hundreds of effector proteins that can be localized in either the apoplasm or cytoplasm of plant cells have been described to be encoded in oomycete genomes (Stassen and Van den Ackerveken, 2011). Within the class of cytoplasmic effectors, the RXLR and Crinkler (CRN) families have been shown to be secreted by the pathogen from infection/nutrition invaginations called haustoria and translocated into host cells thanks to specific amino acid sequences RXLR-EER or LXLFLAK, respectively (Whisson et al., 2007; Schornack et al., 2010). Following infection of sunflower, *P. halstedii* shows intercellular growth and produces haustoria in root, hypocotyl and cotyledon cells (Gascuel et al., 2015). Putative cytoplasmic effectors of RXLR and CRN-type expressed during the interaction were described from transcriptomic and genomic studies (As-sadi et al., 2011; Sharma et al., 2015; Gascuel et al., 2016; Mestre et al., 2016). However, no functional data has been published on *P. halstedii* effector expression *in planta*.

Six putative *P. halstedii* effectors from pathotype 710, five RXLR and one CRN, were selected for functional analyses in sunflower. Their expression was analyzed by q-RT PCR in *P. halstedii* spores and in pathotype 710 infecting sunflower. An *Agrobacterium tumefaciens*- based transient expression assay was set up in sunflower leaves of whole plants to perform functional studies of *P. halstedii* effectors *in planta*. Subcellular localizations of the six *P. halstedii* effectors fused to a fluorescent protein tag were performed for the first time in sunflower. Over-expression of the effector fusions were analyzed in susceptible and resistant sunflower lines carrying one of three different *Pl* loci, *Pl5, Pl6*, or *Pl7*, in order to check their eventual recognition through the triggering of hypersensitive (HR) – like reactions.

## Materials and Methods

### Sunflower Lines and *P. halstedii* Pathotype 710

Three Pl resistance loci (*Pl5, Pl6*, and *Pl7*) confer resistance to *P. halstedii* pathotype 710 in sunflower. Two sunflower Near Isogenic Lines, NIL161R and NIL161S, containing or not the *Pl5* resistance locus were used in this work. The Recombinant Inbred Line (RIL) RIL161 was selected from the INEDI population of RILs (Vincourt et al., 2012) produced by crossing INRA inbred lines XRQ (having the resistant allele of *Pl5* locus on LG13, conferring resistance to pathotype 710) and PSC8 (having the resistant allele of *Pl2* locus on LG8, that does not confer resistance to pathotype 710). The RIL161 line was homozygous for the whole genome except for a heterozygous region of 9.1 cM on LG13 containing the *Pl5* locus. After selfing of the RIL161, 20 progenies were genotyped with markers surrounding *Pl5*. Two homozygous sunflower lines at the *Pl5* locus were selected in the progeny: NIL161S with the *Pl5* susceptible allele from the PSC8 parental line and NIL161R with the resistant allele from the XRQ parental line. This couple of NILs differ genetically only by a region of 9.1 cM of LG13 containing the *Pl5* locus, and have the susceptible allele of *Pl2* locus on LG8.

INRA Inbred lines CAY and GB have no identified *Pl* gene and are susceptible to all *P. halstedii* pathotypes including pathotype 710. CAYRM was obtained by backcrossing the *Pl6* gene from the inbred line YDQ (derived from USDA line HA335) into CAY, and GBRM by backcrossing the *Pl7* gene from the inbred line YEQ (derived from USDA line HA338) into GB. In each case, six back-crosses and two generation of selfing were made to obtain homozygous resistance in a susceptible genetic background.

NIL161R presents type II resistance to *P. halstedii* pathotype 710, as shown for *Pl*5 in other genetic backgrounds, whereas CAYRM and GBRM present type I resistance to 710 (Gascuel et al., 2015). Sporangia and spores of pathotype 710 were collected from sunflower genotype Peredovik [a open pollinated sunflower variety susceptible to all *P. halstedii* pathotypes and used to produce *P. halstedii* sporangia, in a closed growth chamber using the infection method described by Mouzeyar et al. (1993)].

### Plant Growth Conditions

Sunflower seeds germinated for 48 h are soaked in a *P. halstedii* spore solution (10^5^ spores/ml) produced with freshly harvested sporangia from infected and sporulating cotyledons, for 4 h before sowing in flats containing soilless compost. Plants are then grown at 18°C, 16/8 h day/night photoperiod and 80% relative humidity, allowing the development of the disease. Flats are covered for 48 h at 14 dpi to induce sporulation by humidity saturation. *P. halstedii* spores are harvested from infected cotyledons in order to perform DNA and RNA extractions.

For transient expression experiments, sunflower are grown under 16/8 h day/night photoperiod, 75% relative humidity and 22°C. Fourteen days old seedlings were used for infiltration of agrobacteria solution.

For qPCR experiments, seeds were surface sterilized with a bleach solution (2.5% of active chloride) and 0.1% of Tween20 for 20 min. Seeds were rinsed three times with sterile water for 10 min and germinated in Petri dishes containing Campbell medium during 48 h in order to reveal any contamination. Seeds were infected with a spore solution (10ˆ5 spores/ml) freshly harvested from infected cotyledons surfaced sterilized before sporulation for 4 h and then were placed in squared plates with Fahraeus medium in a growth chamber (16/8 h day/night photoperiod, 80% relative humidity, 18°C).

### RNA Extraction and qPCR Experiments

Seedlings were harvested at 3, 7, and 11 dpi, frozen and grinded in 2 ml tubes with a 5 mm steel bead. Total RNA from roots was isolated using NucleoSpin^®^ RNA (Macherey-Nagel). RNA quantity and quality were estimated on some samples using Agilent Bioanalyzer 2100 LabChip and Agilent RNA 6000 Nano kit. An RT reaction was performed with Transcriptor Reverse transcriptase (Roche) on 1 μg of total RNA. Concentration of all samples was estimated using NanoDrop (Thermo Fisher Scientific). Primers were designed in order to amplify 75–150 bp products (Supplementary Table 1). LightCycler 480 SYBR Green I Master was used to achieve qRT-PCR reaction. Eight plants were ground together to avoid different infection stages. Three different biological replicates of eight seedlings were extracted for each infection time, and three technical replicates were performed for qPCR experiments. Mean relative expression was calculated using *P. halstedii RIBS3A* gene encoding 40S ribosomal protein S3a (Supplementary Data 1) as reference gene with three biological and three technical replicates.

### Cloning of Candidate Effectors from *P. halstedii* Pathotype 710

Candidate RXLR effectors were cloned by Gateway cloning (Life technologies). cDNA from *P. halstedii* pathotype 710 spores was used as matrix to amplify putative effectors, with Phusion High-Fidelity Taq DNA polymerase. Specific primers were designed with Primer3 on the coding sequence of each gene (Supplementary Data 1) deleted of its predicted signal peptide, and contained appropriate recombination sequences for Gateway or Golden gate cloning (Supplementary Table 1). PCR products were purified on agarose gel with Wizard SV Gel and PCR Clean-Up System (Promega) and were cloned in a pBIN19 Gateway expression vector pBin19-35S-GW-YFP (Froidure et al., 2010), (kindly provided by Susana Rivas, LIPM Toulouse) in order to create fusion proteins with YFP in N-terminal part of the protein; the effector domain of the protein is free to interact with other molecules and potentially fully functional. CRN37 coding sequence deleted from its first 19 amino acids was fused to a peptide tag GFP under the control of 35S promoter and cloned in one step in the expression vector pCAMBIA-CR1 (Fliegmann et al., 2016) using the cloning method “Golden gate” (Engler et al., 2008, 2009). pCAMBIA-CR1 vector containing GFP under the control of 35S promoter was used as positive control for PhCRN37 transient expression experiments.

### *Agrobacterium*-Mediated Transient Expression Experiments

*Agrobacterium tumefaciens* strain LBA4404 was used for transient expression experiments in sunflower genotypes and GV3101 in *Nicotiana benthamiana*. *A. tumefaciens* transformed strains were grown in a 2 ml preculture overnight in a LB medium containing appropriate antibiotics at 28°C under agitation. Preculture was used to inoculate 15 ml of LB containing antibiotics and cultured overnight under agitation in 50 ml flasks. Bacteria pellets were rinsed and resuspended in agroinfection buffer (10 mM MgCl2, 100 μM acetosyringone) to an OD600 of 0, 5 and incubated 4 h under agitation before infiltration. Leaves were infiltrated by pressure at the abaxial surface in 1 cm^2^ leaf sectors on two leaves per plant using a 1 ml syringe without a needle. pK7WGF2::CRN2 construct (kindly given by S. Kamoun, The Sainsbury laboratory, Norwich, United Kingdom) and pCAMBIA::GFP-PhCRN37 construct in GV3101 strain were prepared and tested in *N. benthamiana* following the same protocol.

### Subcellular Localization of Effector Candidates

Localization assays were performed in the two sunflower lines NIL161S and NIL161R. Three days post infection, leaf disks were harvested, infiltrated with water and observed by a Confocal Leica SP2 AOBS.

### HR-Like Phenotype Notation

Phenotypes of infiltrated sectors were scored at 7, 10, and 13 days post agroinfection on a minimum of 12 plants (24 leaf sectors) per genotype, repeated twice. A cell death index scale (see **Figure 4** in the results section) was used to quantify the responses of infiltrated areas. Non-parametric Wilcoxon test was used for statistical analyses.

### Western Analyses of Fusion Proteins

Infiltrated leaf samples were harvested 72 h post agroinfection. Western blot analyses were made with the Trans-Blot^®^ Turbo^TM^ Transfer System (Bio-Rad) and 10% Mini-PROTEAN^®^ TGX^TM^ Gel. Direct detection of YFP or GFP tag used rabbit anti-GFP antibodies and horseradish peroxidase (HRP) conjugated to a secondary mouse anti-rabbit antibody. Visualization was obtained by chemiluminescent Clarity^TM^ Western ECL Substrate (Bio-Rad) and a G-box (Syngene).

## Results

### Selection of Expressed *P. halstedii* Putative Effectors from Transcriptomic Database

Five putative RXLR and one CRN *Pl. halstedii* effectors were selected for functional analyses in sunflower. They were chosen among the repertoire of expressed effectors identified from the PlasmoparaSp cDNA database^1^ (Mestre et al., 2016). The effector sequences are provided in Supplementary Data 1. The five putative RXLR proteins showed a predicted signal peptide of 17–23 amino acids (SignalP4.1) and putative translocation patterns described in RXLR effectors (**Table 1**). Two of them, PhRXLR08 and PhRXLR31 have only a DEER domain but were predicted as RXLR effectors in the *P. halstedii* strain recently sequenced (Sharma et al., 2015; **Table 1**). The three others had either exact or very close RXLR-EER motifs (RMLQ-EDR for PhRXLR14) (**Table 1**). PhRXLR02 and PhRXLR03 showed homology (blastp *e*-values < 10E-5) with a predicted RXLR effector from *Phytophthora infestans*, PITG_04354 (**Table 1**). PhRXLR02 and PhRXLR03 are two related proteins sharing 83% identity (*e*-value < 10E-43) previously detected as part of the same blastn cDNA RXLR family, as defined in Mestre et al. (2016), and located on the same genomic scaffold (Gascuel et al., 2016). Altogether, the five RXLRs belonged to four different blastn cDNA families, suggesting that the encoded proteins might have diverse functions (Mestre et al., 2016).

**Table 1:** Description of the six *Plasmopara halstedii* putative effectors used for functional studies.

Effector name	cDNA cluster name in PlasmoparaSp database^∗^	Predicted peptide size	SignalP prediction	Effector conserved motifs	BlastP best hit in *P. halstedii* proteome^+^	Effector protein name^+^	% identity	% subject	*e*-value	BlastP best hit in *P. infestans* proteome	% identity	% subject	*e*-value	*P. infestans* protein annotation
PhRXLR02	Plhal002956	114	1_19	RSLR -EER	nd	nd				PITG_04354	32.81	40	6E-06	Putative secreted RxLR effector peptide protein
PhRXLR03	Plhal004047	100	1_19	RSLR -EER	nd	nd				PITG_04354	34.38	40	5E-06	Putative secreted RxLR effector peptide protein
PhRXLR08	Plhal004370	151	1_20	DEER	CEG48134.1	PHALS_05607	100	100	7E-114	nd				
PhRXLR14	Plhal020884	274	1_17	RMLQ-EDR	CEG43625.1	nd	100	85	5E-179	nd				
PhRXLR31	Plhal003241	199	1_23	DEER	CEG35747.1	PHALS_00082	100	90	3E-123	nd				
PhCRN37	Plhal000548	418	No	LTLYLAKK-HVLVELP	CEG36555.1	nd	98.56	100	0.0	PITG_18503	56.72	75	4E-131	Crinkler (CRN) family protein

*^∗^httpsr://www.heliagene.org/PlasmoparaSpecies (Mestre et al., 2016). ^+^corresponding protein names (% identity > 90%) in the *P. halstedii* proteome published by Sharma et al. (2015). Nd, not detected; e-value threshold, 1E-05. Searches for best hit sequences were performed using BlastP on oomycete proteomes indicated (*E*-value < 10e-5). *P. infestans* is *Phytophthora infestans*.*

The *P. halstedii* putative CRN effector, PhCRN37 showed the predicted translocation motif LTLYLAK fitting well with the CRN consensus sequence LXLFLAK of oomycete CRN (Haas et al., 2009; Schornack et al., 2010), LXLYLAK being highly represented in *P. halstedii* expressed CRN (11 cases out of a total of 54 analyzed) (Mestre et al., 2016). PhCRN37 showed no signal peptide according to different SignalP prediction software (V2, V3, and V4.1) but its N-terminal domain fitted well with the N-terminal domain of *Phytophthora infestans* CRN (Haas et al., 2009), and with those of *P. halstedii* and *P. viticola* CRN according to the weblogo conservation profiles (Mestre et al., 2016). These observations suggest that a signal peptide not predicted by the software used could be present in PhCRN37. PhCRN37 had a C-terminal domain of 318 amino acids localized after HVLVELP, the other important motif commonly found in oomycete CRN, showing 83% identity (*e*-value 1E-19, see alignment in **Figure 3A**) with the DXZ domain described in *Phytophthora infestans* CRN2 (Haas et al., 2009). The greatest homology of PhCRN37 C-terminal region was found in the necrotroph oomycete *Pythium ultimum* (blastp *e*-value = 0, Mestre et al., 2016), but it had also close homologs (*e*-values < 10e-80, corresponding to >60% identity and >90% coverage of the query sequence) in biotroph *Pseudoperonospora cubensis* and hemibiotroph oomycetes *Phytophthora infestans, Phytophthora sojae*, and *Phytophthora ramorum* (Mestre et al., 2016).

### Expression of Candidate Effector Genes in Infected Sunflower

**FIGURE 1:**
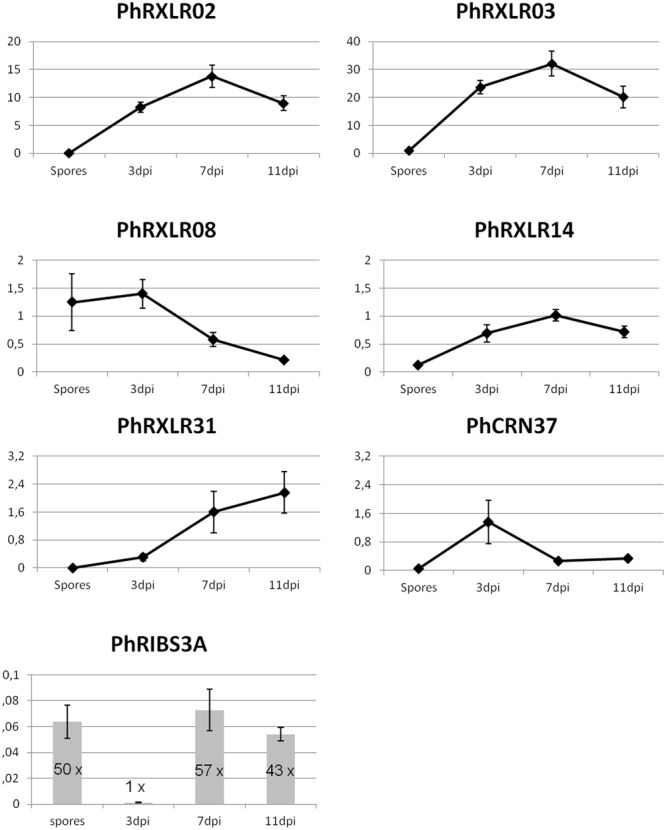
**Q-RT PCR gene expression patterns of six putative effector genes and a control gene, *PhRIBS3A*, in *P. halstedii* spores and in infected sunflower seedlings at 3, 7, and 11 days post inoculation (dpi).** Relative expression values of *P. halstedii* effector genes compared to housekeeping gene *PhRIBS3A* were calculated by the ΔCT method. *PhRIBS3A* expression ratio was normalized by the mean of plant constitutive genes by the ΔCT method. Standard error was calculated for each point for three biological repetitions and three technical repetitions.

To further confirm that the *in silico* predicted effector genes are really expressed in the pathogen infecting sunflower plants, q-RT PCR experiments were performed in a suspension of *P. halstedii* spores and in susceptible sunflower seedlings at 3, 7, and 11 days post infection (dpi) with the *P. halstedii* pathotype 710. Expression of *P. halstedii RIBS3A* (*PhRIBS3A*) housekeeping gene encoding for the Ribosomal protein S3A was used to monitor *in planta* pathogen growth and to normalize effector gene expression (**Figure 1**). *PhRIBS3A* transcripts were detected at high levels in free spores before inoculation (mean Ct = 29 ± 0.3). In inoculated susceptible sunflower plants, *PhRIBS3A* quantification was hardly detectable at 3 dpi (mean Ct = 36 ± 0.5) probably because of low *in planta* pathogen content at that stage, but increased at 7 and 11 dpi, indicative of *in planta* pathogen growth. Compared to 3 dpi, it increased by 57-fold at 7 dpi (mean Ct = 30.7 ± 1) and by 43-fold at 11 dpi (mean Ct = 31 ± 0.5), stages corresponding to an important colonization of the plant tissues, 11 dpi being at the onset of sporulation (Gascuel et al., 2015).

After normalization with *PhRIBS3A*, all effector genes tested except *PhRXLR08*, showed a low or non-significative expression in spores, and induction of their expression in infected sunflower tissues compared with spore suspensions (**Figure 1**). *PhRXLR02* and *PhRXLR03* genes showed the highest induction levels, showing at 3 dpi 8.3 ± 0.9 and 23.7 ± 2.4 relative expression values (rev), respectively, reaching a peak of expression at 7 dpi (13.8 ± 2.0 and 32.1 ± 4.4 rev), and decreasing at 11 dpi close to 3 dpi levels (**Figure 1**). *PhRXLR14* gene showed a similar but lower induction pattern as *PhRXLR02* and *PhRXLR03*, with a maximum at 7 dpi of 1.0 ± 0.1 rev. *PhRXLR08* and *PhRXLR31* genes displayed different induction patterns from previous RXLRs. They showed opposite patterns one from another, (i) *PhRXLR08* being expressed in spores and at 3 dpi at similar expression levels (1.2 ± 0.5, and 1.4 ± 0.3 rev, respectively) which decreased at 7 and 11 dpi, (ii) *PhRXLR31* being weakly induced at 3 dpi (0.3 ± 0.1 rev) and increased gradually at 7 dpi (1.6 ± 0.6 rev) and 11 dpi (2.2 ± 0.6 rev). *PhCRN37* gene expression peaked early at 3 dpi (1.4 ± 0.6 rev) and decreased thereafter to low levels (0.3 ± 0.1 rev at 7 and 11 dpi) (**Figure 1**). In conclusion, two expression profiles were observed: (i) an early expression pattern for two effector genes (*PhRXLR08* and *PhCRN37*), (ii) an increased expression from spores to early and late infection stages for the four other effector genes (*PhRXLR02, PhRXLR03, PhRXLR14*, and *PhRXLR31*), *PhRXLR02* and *PhRXLR03* showing the highest expression levels. These 6 *P. halstedii* effector genes expressed in inoculated sunflower were cloned in an *Agrobacterium* binary vector for further functional studies.

### Subcellular Localization of Six *P. halstedii* RXLR and CRN Putative Effectors

**FIGURE 2:**
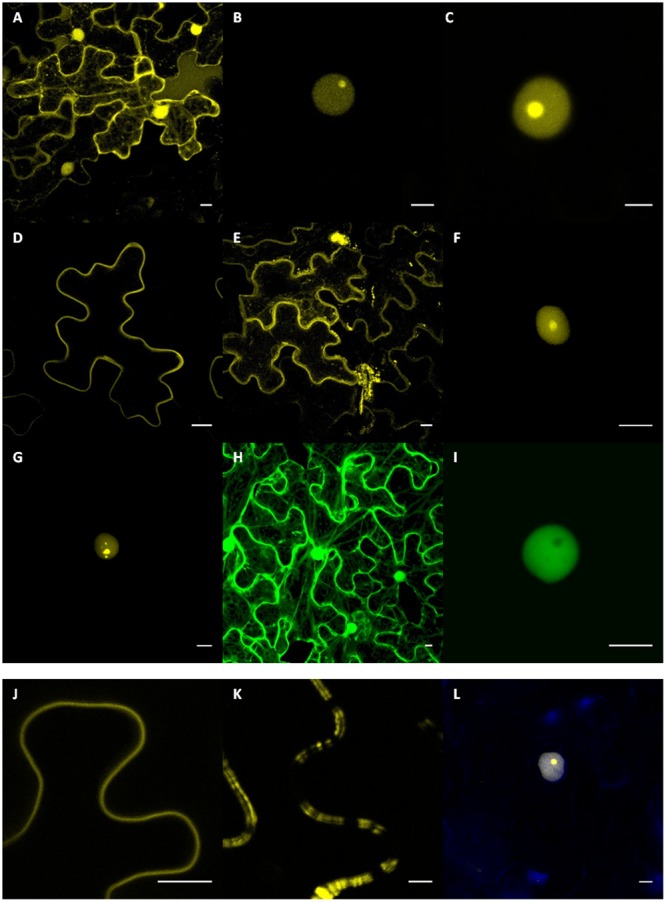
**Subcellular localizations of PhRXLR and PhCRN fusion proteins in sunflower leaf tissues.** Sunflower **(A–K)** and *Nicotiana benthamiana* **(L)** leaf tissues showing expression of the YFP-RXLR and GFP-CRN fusion protein genes driven by the 35S promoter after *Agrobacterium*-transient expression. Results presented **(A–K)** are from susceptible NIL161S sunflower line. Scale bar = 10 μm. **(A)** Subcellular localization of the YFP protein in the nucleus and cytoplasm. Nuclear localization of PhRXLR02 **(B)** and PhRXLR03 **(C)** fused to YFP showing intense nucleolar fluorescence. Representative images of *in planta* plasma membrane-like localization of PhRXLR08 **(D)** and PhRXLR14 **(E)** fusion proteins. **(F,G)** PhRXLR31-YFP localizes at the nucleus, showing a dynamic subnuclear localization varying from nucleolar localization to speckles-like structures. **(H)** Subcellular localization of the GFP protein in the nucleus and cytoplasm. **(I)** PhCRN37-GFP showed a nucleoplasmic localization. Detail of the plasma-membrane like localization of PhRXLR08 **(J)** and PhRXLR14 **(K)**. **(L)** DAPI staining of nuclei expressing PhRXLR03 in transiently transformed *N. benthamiana* epidermal cells.

The coding sequence of each *P. halstedii* RXLR effector, deleted from its predicted signal peptide was fused in frame to the C-terminal end of the fluorescent YFP coding sequence for *in planta* subcellular localization studies. The signal peptide of the oomycete protein is supposed to be cleaved off upon secretion from the oomycete as for other eukaryotes, and therefore it should no longer be present in the protein that goes to the plant cell *via* the translocation motives (Bozkurt et al., 2012; Fawke et al., 2015). To adopt a similar cloning strategy, GFP-PhCRN37 was built by removing the 17 first amino acids of *PhCRN37* predicted protein corresponding to a conserved region in several *P. halstedii* CRN (Mestre et al., 2016). These constructs were placed under the control of the 35S promoter in an *Agrobacterium* binary vector. Following *Agrobacterium*-mediated transient expression, the fluorescence of YFP or GFP was localized in cytoplasm and nucleus of the sunflower cell when expressed alone (**Figures 2A,H**), as observed in *Nicotiana benthamiana* (not shown).

YFP-PhRXLR02 and YFP-PhRXLR03 fusion proteins localized to the sunflower cell nucleus, and mainly in nucleolus where it gave a brighter signal, suggesting a higher concentration of the fusion protein in the nucleolus (**Figures 2B,C**). YFP-PhRXLR31 fusion protein localized also to sunflower nucleus (**Figures 2F,G**) but different localizations could be observed in different nuclei revealing a dynamic subnuclear localization for this protein. The fluorescence localized in nucleolus and nucleoplasm of most nuclei (**Figure 2F**), but in some nuclei, a punctuated localization in uncharacterized bodies could be observed (**Figure 2G**). GFP-PhCRN37 fusion localized also to the sunflower cell nucleus, but mainly in nucleoplasm as no fluorescence was observed in the nucleolus (**Figure 2I**). DAPI staining was performed to confirm nuclear localizations (**Figure 2L** and data not shown).

YFP fused to PhRXLR08 or to PhRXLR14 localized to the cell border, probably at the plasma membrane (**Figures 2D,E**). YFP-PhRXLR14 localized also in cytoplasm and in some undetermined cell bodies (**Figure 2E**). However, unlike YFP-PhRXLR08 (**Figure 2J**), YFP-PhRXLR14 showed a double and irregular pattern at the cell outline (**Figure 2K**), typical of some plasma-membrane located proteins (Jo et al., 2011; Trehin et al., 2013). Co-localization experiments using appropriate constructs with known localization in cell compartments would be helpful to precise the localization of *P. halstedii* effector fusions.

Different subcellular localization patterns were observed for the six effector fusions, but no difference was observed between the resistant and susceptible sunflower genotypes tested. Subcellular localizations in *N. benthamiana* leaves were identical to those observed in sunflower leaves. Protein integrity and molecular weight of the protein fusions were checked by western blot analyses on sunflower leaf proteins (data not shown).

### Four *P. halstedii* Putative Effectors Induce HR-Like Responses in Resistant Sunflower Lines But Not in *N. benthamiana*

**FIGURE 3:**
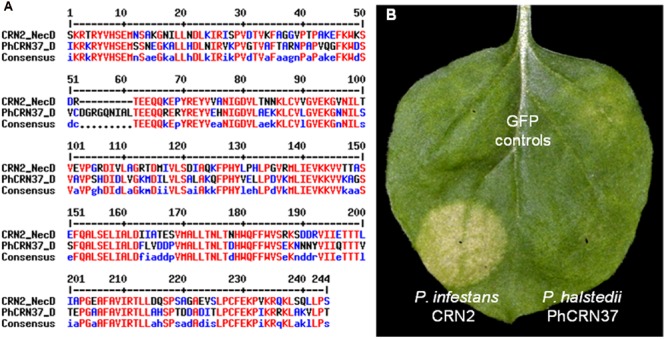
***Plasmopara halstedii* CRN effector, PhCRN37, does not exhibit a necrosis phenotype in *N. benthamiana.* (A)** Alignment of CRN2 necrotic domain (CRN2_NecD, 173–407 amino acids) of *Phytophthora infestans* CRN2 protein with the corresponding domain of PhCRN37 (PhCRN37_D, 126–369 amino acids). **(B)** Unlike CRN2 effector from Phytophthora infestans, PhCRN37 does not induce cell death after infiltration in *N. benthamiana* leaf. Picture was taken at 4 days post infiltration.

Transient expression assays performed on *N. benthamiana* with the six *P. halstedii* effector fusions gave no HR-like responses. This was not really surprising since (i) most of these effectors are specific to *P. halstedii* (**Table 1**), (ii) *P. halstedii* is a pathogen restricted only to some Asteraceae species and does not infect Solanaceous species such as *N. benthamiana*. However, PhCRN37 is closely related to *Phytophthora infestans* CRN2 (blastp *e*-value 4E-131, 48% identity, **Table 1**) especially in its C-terminal 234 amino-acid region defined as domain DXZ (Haas et al., 2009), showing 64% of identical amino acids (**Figure 3A**). CRN2, as well as the CRN2 DXZ region was shown to be sufficient to induce cell death when expressed in *N. benthamiana* cells (Haas et al., 2009; **Figure 3B**). Unlike CRN2, PhCRN37 was not able to induce cell death in *N. benthamiana*, suggesting that the polymorphism observed in PhCRN37 was sufficient to prevent cell death induction in those cells (**Figure 3B**).

Transient expression assays were then performed in sunflower plant cells. We selected a couple of sunflower near-isogenic lines (NIL) and introgression lines (xxRM) carrying different *Pl* resistance loci to pathotype 710, in order to cover most of sunflower genetic resistance to *P. halstedii* pathotype 710, and to improve likelihood of recognition of the effectors tested. Inbred sunflower lines NIL161R, CAYRM, and GBRM have *Pl5, Pl6*, and *Pl7* loci, respectively, and are resistant to *P. halstedii* pathotype 710, whereas susceptible lines NIL161S, CAY, and GB have, respectively, the same genetic backgrounds as the resistant lines, but carry the susceptible alleles to pathotype 710 of *Pl* loci.

**FIGURE 4:**
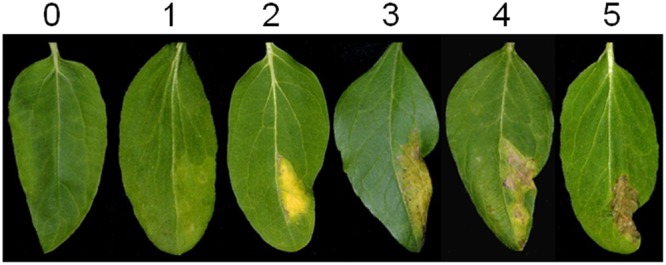
**Scoring scale of sunflower leaves used in *Agrobacterium*-transient experiments**.

The phenotypical effect of transient overexpression of each effector fused to GFP or YFP was estimated quantitatively in sunflower leaves using a rating scale from zero (no cell death) to 5 (a dry necrosis extending all over the infiltrated area) (**Figure 4**). We considered that scores of 4 (large sectors of dry necroses inside the infiltrated area) to 5 corresponded to HR–like responses.

**FIGURE 5:**
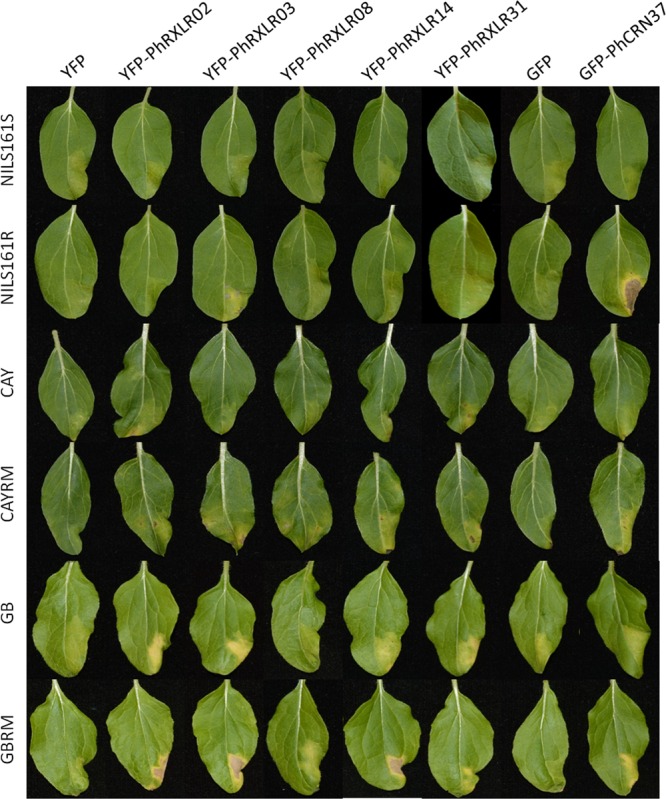
**Recognition of *P. halstedii* RXLR and CRN effectors in resistant sunflower genotypes by HR-like responses**. Phenotypes observed in sunflower leaves of six different genotypes (susceptible genotypes: NIL161S, CAY, and GB, and resistant genotypes to pathotype 710 of *P. halstedii*: NIL161R, CAYRM and GBRM; see text for explanations) after *Agrobacterium*-transient expression of the different YFP-PhRXLR, or the GFP-PhCRN37 fusions driven by the 35S promoter. Pictures were taken 11 days post infiltration.

**FIGURE 6:**
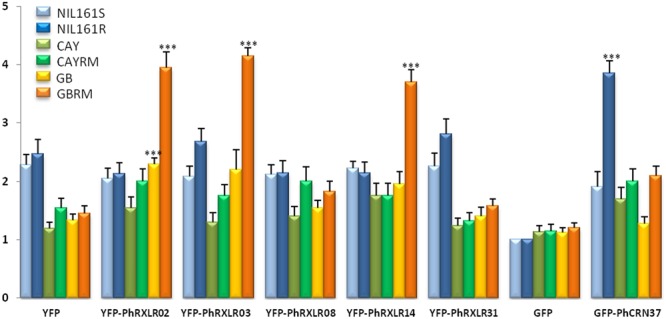
**Mean scores estimated on sunflower leaf sectors 11 days post infiltration with the *P. halstedii* YFP-RXLR and GFP-CRN effector fusions.** Twenty four leaf sectors were scored in two representative independent experiments. Statistical analysis was performed with Wilcoxon test. Asterisks indicate significant differences with the control constructs in the same sunflower genotype (YFP for PhRXLRs and GFP for PhCRN37), calculated for the two biological replicates. ^∗∗∗^*P*-value < 0.001.

Four *P. halstedii* effector fusions (GFP-PhCRN37 and three RXLRs: YFP-PhRXLR02, YFP-PhRXLR03, and YFP-PhRXLR14) among the six tested induced cell death at a significantly higher level than GFP or YFP alone in at least one sunflower genotype (**Figures 5** and **6**). PhRXLR02, PhRXLR03, and PhRXLR14 fused to YFP induced HR-like responses when expressed in GBRM leaves containing *Pl7* resistance locus (mean score around 4), but only chlorosis in the corresponding susceptible parent GB (mean score at 2 and below) (**Figures 5** and **6**). The cell death was confluent at 10 days post agroinfection (dpa) in this genotype. GFP-PhCRN37 induced an HR-like response, visible from 7 dpa, only in NIL161R, which carries *Pl5* resistance locus, and not in the corresponding susceptible line, NIL161S (**Figure 5**). No confluent HR-like responses were observed in the infiltrated areas of the line CAYRM, containing the *Pl6* locus, with any of the effectors tested, nor in the corresponding susceptible isogenic line CAY. Necrotic dots were sometimes detected in CAYRM leaves infiltrated with YFP-PhRXLR02, YFP-PhRXLR03 and YFP-PhRXLR31 (**Figure 5**).

Scoring responses of the infiltrated leaf areas were quantified at 11 dpa in 24 sunflower leaf sectors per genotype (**Figure 6**). For the four effectors inducing visible and confluent cell death (**Figure 5**), the mean levels were significantly higher (*P*-value < 0.001) when they were expressed in the leaves of resistant sunflower lines compared to corresponding susceptible lines, suggesting that they could be recognized by the *Pl* locus present in the resistant plant. PhRXLR02, PhRXLR03, and PhRXLR14 YFP fusions induced HR-like cell death in response to the *Pl7* locus introgressed in GBRM, whereas GFP-PhCRN37 was recognized by the *Pl5* resistance locus functional in NIL161R but not in NIL161S.

## Discussion

Throughout this work, we performed the functional analysis of six putative effectors of *P. halstedii*, sunflower downy mildew. Expression experiments showed that some of these candidate effectors are highly induced during infection, suggesting their potential importance in host colonization process by the oomycete. However, induction levels measured *in planta* were variable according to the effectors tested. We had noticed previously that expression of putative *P. halstedii* effector genes showed considerable variability between different infected tissues of sunflower seedlings, for example between roots and hypocotyls, 3 dpi (data not shown). This variability could be due to the difficulty in measuring real *in planta* infection levels and synchronizing developmental stages of *P. halstedii* during its interaction within the plant. In the expression experiments presented here, we therefore chose to extract RNA from whole infected seedlings at different time points, in order to minimize the distribution bias due to uneven *in planta P. halstedii* development. We also normalized effector gene expression levels with a constitutive *P. halstedii* gene encoding a ribosomal protein, used as an *in planta P. halstedii* quantification.

The only way to perform *P. halstedii* infection in sunflower is to inoculate germinated seeds at a given spore concentration, then plant them in soil, and after 10–12 days growth, cover the seedlings to increase relative humidity in order to provoke sporulation on cotyledons or leaves 2 days later in the case of downy mildew susceptibility. The number of successful infections with entry of the pathogen into the root cannot be easily measured quantitatively, other than by counting hyphae under the microscope from cross sections of roots or hypocotyls stained with lactophenol blue (Gascuel et al., 2015). Internal growth curve (IGC) experiments widely used to quantify numbers of *in planta* pathogenic bacteria cannot be adapted to *P. halstedii*, due to its obligate biotrophic character, and its inability to grow *in vitro*. The progression of the pathogen within the seedling is also difficult to estimate accurately both spatially and during time, although there seems to be a well organized infection pattern, the pathogen hyphae going straight from roots to aerial parts of the plant where sporulation occurs (Gascuel et al., 2015).

*Plasmopara halstedii* is an obligate biotroph oomycete showing no necrotrophic phase of infection. Three PhRXLR effectors tested peaked at 7 dpi, i.e., a few days before sporulation and which could still be considered as early stages of infection for *P. halstedii*. PhRXLR31 was maximally induced at later stages, close to sporulation and could correspond to a virulence factor necessary for pathogen colonization and disease. The expression pattern of PhCRN37, with its maximal induction at 3 dpi, resembles more closely that of a classical avirulence gene. However, to our knowledge, no *senso stricto* avirulence function for a CRN effector has been shown. *P. viticola* RXLR expression patterns peaked as early as 48 h post-infection, probably because of the direct leaf disk dipping method used for grape downy mildew inoculation (Mestre et al., 2012; Yin et al., 2015).

Although *Agrobacterium*-mediated transient expression of genes into leaves of entire plants has been described for many plants, such a technique did not exist for sunflower, and in the Asteraceae family, it was described for lettuce (Krenek et al., 2015). Stable transformation of sunflower by *A. tumefaciens* is still difficult, with poor efficiency and is restricted to specific sunflower lines (Knittel et al., 1994; Malone-Schoneberg et al., 1994; Burrus et al., 1996; Lucas et al., 2000). Transient transformation of detached sunflower leaves infiltrated *under vacuo* with *A. tumefaciens* was proposed in order to overcome structural leaf problems and low bacterial transformation efficiencies (Manavella and Chan, 2009; Jung et al., 2014). The tedious but successful adaptation of these protocols to sunflower leaves of entire plants was, for us, a prerequisite for functional studies of *P. halstedii* effectors. We therefore tested several *A. tumefaciens* strains with various virulence levels carrying a GUS (*uidA* gene) – intron construct, on different sunflower genotypes in order to improve transformation efficiency. We found that the *A. tumefaciens* strain LBA4404 was the best compromise between a good cell transformation level and a limited stress infiltration in leaves of different sunflower lines. Setting up this test was instrumental to achieving subcellular localization of *P. halstedii* effectors in its host plant and essential to testing their eventual recognition in resistant sunflower genotypes. Since the host range of *P. halstedii* is restricted to only a few *Helianthus* species, transient expression of *P. halstedii* effectors in *N. benthamiana* or any other plant easy to transform would not reply to questions related to host specificity and effector recognition. Using this protocol, we were able for the first time to initiate functional analyses of putative sunflower downy mildew effectors.

Three *P. halstedii* effectors fused to the YFP fluorescent protein, PhRXLR02, PhRXLR03, and PhRXLR31, were localized in the sunflower cell nucleus, and mainly in nucleolus. GFP-PhCRN37 showed also nuclear localization but was excluded from the nucleolus. PSORT predictions^2^ suggested nuclear localization for PhRXLR02, 03 and PhCRN37 fusion proteins because of presence of nuclear localization signals that were not detected in PhRXLR31. Forty-nine RXLR effectors (HaRXLs) of Arabidopsis downy mildew, *H. arabidopsidis*, were studied functionally *in planta* (Caillaud et al., 2012). Thirty-three percent of HaRXLs were exclusively found in the *A. thaliana* cell nucleus, and 22% showed nucleolar localizations (Caillaud et al., 2012). Nuclear localization of oomycete CRN effectors is even more frequent, for example all CRNs identified in *Phytophthora capsici* were localized in the *N. benthamiana* cell nucleus (Stam et al., 2013a,b). Nuclear localization in *N. benthamiana* was reported as essential for the necrotic function of CRN effectors from *Phytophthora infestans* and *Aphanomyces euteiches* (Schornack et al., 2010). Interestingly, PhCRN37 from *P. halstedii* and CRN2 from *Phytophthora infestans*, which both have a CRN DXZ domain presented the same subcellular localization in the nucleus with nucleolus exclusion in *H. annuus* and *N. benthamiana*, respectively (Schornack et al., 2010 and our data). High sequence conservation and similar localization in both plants could *a priori* suggest that their role in pathogen virulence might be similar; we showed, however, that at least the induced cell death activity in *N. benthamiana* is lost in PhCRN37 compared to *Phytophthora infestans* CRN2.

The different intranuclear localizations of effectors could also give us information about their possible functions. The plant cell nucleus can be divided into two prominent compartments, the nucleolus and the nucleoplasm. The nucleolus is mainly involved in transcription of ribosomal RNA genes, maturation of precursor rRNA, and assembly of ribosomal subunits (Shaw and Brown, 2012), but also in other functions including transport, splicing of RNA and transcriptional gene silencing (Pontes and Pikaard, 2008; Rodor et al., 2010). Gene transcription occurs in the nucleoplasm, which contains most DNA material, transcription factors, and proteins involved in DNA and RNA synthesis. Like bacterial TAL effectors, nucleoplasm localization of oomycete effectors like PhCRN37 could promote pathogen virulence by activating or suppressing expression of host genes, respectively, involved in plant susceptibility or resistance. Nucleolar localization suggests that *P. halstedii* effectors PhRXLR02, PhRXLR03, and PhRXLR31 may target rRNA and ribosome biosynthesis, thus acting on protein translation of plant defense genes as was hypothesized for *H. arabidopsidis* RXLRs (Caillaud et al., 2012). The dynamic subnuclear localization of the YFP-PhRXLR31 still raises questions. Although YFP-RXLR31 is mainly located in nucleolus and nucleoplasm, the fusion protein is also observed in certain nuclei in uncharacterized and unstable bodies, known as speckles by certain authors. Nuclear speckles have been described as dynamic structures showing a punctate pattern within the nucleus of mammalian cells. They change in composition over time and co-localize with actively transcribed chromatin regions (Lamond and Spector, 2003). In plants, nuclear speckles correspond to storage and assembly areas that procure splicing factors to active transcription sites; according to speckle size, they are associated with activation or inhibition of transcription or splicing (Reddy et al., 2012). The localization of YFP-RXLR31 in speckles could suggest that PhRXLR31 might be involved in such processes leading to host plant gene regulation.

Another major cell compartment targeted by RXLR effectors is the membrane network (26% of HaRXLs) including plasma membrane for 6% (Caillaud et al., 2012). Two *P. halstedii* putative effectors fused to YFP, PhRXLR08, and PhRXLR14, were localized to the sunflower cell border, probably at the plasma membrane. YFP-PhRXLR08 was only localized there, with a continuous labeling, whereas YFP-PhRXLR14 showed an irregular labeling at the cell border. The double and irregular labeling observed for YFP-PhRXLR14 was shown for plasma membrane-localized proteins from rice and *A. thaliana* (Jo et al., 2011; Trehin et al., 2013). YFP-PhRXLR14 showed in addition to the cell border labeling in the cytoplasm and in undetermined bodies that might be peroxisomes, as predicted by PSORT localization (*P* = 0.64), but more probably because of their unclear appearance, vesicles from the secretory pathway (known as late endosomes).

Because trans-membrane proteins like pathogen recognition receptors (PRRs) constitute the first level of microbe recognition in plants in Pattern Triggered Immunity (PTI), membrane localized effectors might target PRRs and be involved in disrupting plant immunity. Oomycete RXLR effectors could directly target PRR associated proteins to inhibit induction of plant immune signals (Caillaud et al., 2012). The role of effectors targeting the plasma membrane in pathogen virulence was shown with Arabidopsis lines expressing HaRxLR77 which were more susceptible to *H. arabidopsidis* than wild type (Caillaud et al., 2012). HaRxLR77 was suggested to prevent exocytosis of defense components or endocytosis of PRR that activate PTI responses. The AVRblb2 RXLR effector from *Phytophthora infestans* is also plasma membrane localized, and interferes also with vesicle trafficking to prevent secretion of host cysteine protease C14 (Bozkurt et al., 2011). *P. halstedii* produces haustoria in the sunflower cell (Gascuel et al., 2015). It would be interesting to investigate *P. halstedii* effector localization in cells presenting haustoria, as was done for RXLR effectors from model oomycetes (Bozkurt et al., 2011; Caillaud et al., 2012).

Numerous RXLR genes from oomycete pathogens were shown to be involved in the triggering of HR and resistance to infection in the presence of a specific resistance plant gene, illustrating the “gene-for-gene hypothesis” in oomycetes (for reviews, Hein et al., 2009; Fawke et al., 2015). This perception mechanism is based on plant resistance (R) proteins that confer indirect or direct recognition of specific pathogen avirulence (Avr) proteins. In the sunflower –*P. halstedii* interaction, no such gene-for-gene interaction has been described yet, probably because of the difficulty in studying this pathosystem (Gascuel et al., 2015), and the recent availability of molecular data for the two partners (As-sadi et al., 2011; Sharma et al., 2015; Gascuel et al., 2016^3^; Mestre et al., 2016). Complexity and size of the sunflower genome, together with lack of reverse genetics techniques in both partners have hampered validation of candidate genes involved in resistance.

For these reasons, we chose the *Agrobacterium*-mediated transient expression method to test possible recognition of *P. halstedii* effector genes from pathotype 710, by sunflower lines carrying three different resistance loci to this pathotype. We were able to use sunflower lines that have been introgressed with two *Pl* regions, *Pl6* and *Pl7*, and NIL with or without the *Pl5* locus, in order to eliminate effects of genetic background variations outside the *Pl* regions. None of the six *P. halstedii* effectors tested induced HR- like responses in the susceptible lines, and the stress response induced by agroinfection, as indicated by responses to YFP and GFP alone, remained at a low level for the six resistant or susceptible genotypes. This result makes it possible to validate the HR-like responses obtained with four effectors, three RXLR and one CRN that were recognized by one of the resistant lines.

Hypersensitive-like responses were very clear and confluent at 10 days post agroinfection and easily distinguishable from a yellowish background observed with the control construct on some sunflower lines. However, decoloration and spotted necrotic areas were visible much earlier, at 3 or 6 days post infiltration, respectively, in the leaf sectors that will show later on confluent HR. In comparison, HR triggered in *N. benthamiana* by other RXLR or CRN effectors could be visible at 4 days after agronfection but were usually shown after 6–8 days (Vleeshouwers et al., 2008; Haas et al., 2009; Tian et al., 2011; Wang et al., 2011; Bozkurt et al., 2012). In other species, such as lettuce, 6 days were necessary (Stassen et al., 2013). Using *PsojNIP1* encoding a necrotic –like protein of *Phytophthora sojae* (Cabral et al., 2012), we observed that sunflower needs at least 2 more days to get a clear dry HR-like response compared to *N. benthamiana*. This could be due to a lower transformation cell rate in sunflower compared to *N. benthamiana*, and probably also to lower expression levels of the construct in sunflower.

Three RXLR effector fusions were recognized by the resistant line GBRM carrying *Pl7* locus, (i) PhRXLR02 and PhRXLR03 that share sequence similarity and are nuclear localized, and (ii) PhRXLR14 that is very different in sequence and localized at the cell border. We suggest the hypothesis that a protein encoded by a gene present in the *Pl7* introgressed region in GBRM would recognize both PhRXLR02 and PhRXLR03. In the guard model (Jones and Dangl, 2006) it was shown that the R protein is not the only plant partner involved in the interaction and that the direct recognition of the Avr protein is often mediated by a guardee. The *A. thaliana* RPM1 R protein can enable dual specificity disease resistance of two distinct bacterial avirulence proteins, AvrB and AvrRpm1 and trigger HR with both (Grant et al., 1995). RPM1 was later defined as being the R guard that recognizes different modifications performed on a common guardee RIN4 (Grant et al., 2006). In the case of PhRXLR14, which has a different cellular localization from PhRXLR02 and 03, we can propose that it is recognized by another gene present also in the introgressed GBRM region. Unfortunately, the *Pl7* region is poorly documented in the literature (Vear et al., 1997). The closely linked *Pl6* region has been studied in more detail and has revealed the complexity of sunflower genomic regions involved in downy mildew resistance. According to Bouzidi et al. (2002) the *Pl6* locus is suspected to span several megabases of LG8 and to contain at least 11 functional *Pl* genes conferring resistance to several pathotypes of *P. halstedii*. In this region, these authors mapped specific markers derived from 13 different RGAs belonging to the TIR-NBS-LRR class of plant resistance genes (Bouzidi et al., 2002). A more recent study by positional cloning proposed in this region a TIR-NBS-LRR gene candidate for a *Pl* resistance gene that would confer resistance to pathotype 300 (Franchel et al., 2013).

The *P. halstedii* PhCRN37 induced HR-like responses only in the NIL carrying the *Pl5* locus, introduced from the sunflower line XRQ, that confers resistance to pathotype 710 (As-sadi et al., 2011). The region of the *Pl5* locus also contains a large number of RGAs (Radwan et al., 2004). Our results suggest that a *P. halstedii* CRN effector might be recognized in a specific resistant host and not in the susceptible near-isogenic line, nor in the non-host plant *N. benthamiana*. Until now, the role of this ancient class of effectors found in every oomycete species (Schornack et al., 2010), was thought to induce cell death or to suppress plant immunity responses (Torto et al., 2003; Haas et al., 2009; Liu et al., 2011; Li et al., 2016). We can speculate that, according to its early expression pattern and its recognition specificity, PhCRN37 might act as an avirulence gene that would be recognized by one of the numerous RGAs present in sunflower *Pl5* locus region (Radwan et al., 2004).

The function of avirulence gene for *P. halstedii* effectors will be difficult to prove definitely, as no reverse genetics technique is available for this pathogen, and no sunflower cognate resistance gene has been cloned. For some effectors, we could benefit from the polymorphic alleles present in pathotypes showing divergent virulence patterns and test them with *Agrobacterium*-mediated transformation on appropriate lines (Gascuel et al., 2016).

For the two effector genes not recognized, we have no clue whether they really play a role in the pathogenicity of *P. halstedii*. Other *Pl* loci (*Pl Arg, Pl16* and *Pl13*) mapped on LG1 confer resistance to pathotype 710 and could have been tested. Unfortunately, no introgressed lines were available. Suppression of Pattern Triggered Immunity (PTI) is a good marker to test the role of a given effector in virulence. It would be interesting to test whether *P. halstedii* RXLR or CRN effectors are able to suppress PTI induced by *Phytophthora infestans* infestin INF1, for example, as does the *Phytophthora infestans* Avr3A RXLR effector (Bos et al., 2006) and more recently, several RXLR from *P. viticola* (Xiang et al., 2016).

Effectors can be exploited in breeding against biotrophic pathogens. Functional genomic approaches (known as “effectoromics”) can be performed using effectors for probing plant germplasms to detect new *R* genes (Vleeshouwers and Oliver, 2014). Effectors can provide a tool to accelerate resistance gene cloning as generation of stable transformations can be by-passed by quick functional assays based on transient expression. This strategy has already been applied to *Phytophthora infestans* and potato and led to the characterization of novel resistance genes in non-cultivated species of potato (Vleeshouwers et al., 2008). More recently, a RXLR-variant GKLR effector of the obligate biotroph oomycete causing lettuce downy mildew, *B. lactucae*, was shown to be recognized in lettuce cultivars containing the *Dm2* gene, and proposed to be a candidate *Avr2* avirulence gene (Stassen et al., 2013). Similar strategies applied to a larger set of *P. halstedii* effectors and various sunflower germplasms should contribute to determine *P. halstedii* avirulence genes and to increase and discover novel resistances that can be exploited in breeding. With appropriate segregating sunflower populations, it should also in term accelerate the map-based cloning of sunflower downy mildew resistance genes.

## Author Contributions

Conceived and designed the experiments: QG, LB, and LG. Performed the experiments: QG, LB, and YP. Analyzed the data: QG, LB, YP, and LG. Contributed reagents/materials/analysis tools: NB, SM, and FV. Wrote the paper: QG, LB, and LG.

## Conflict of Interest Statement

The authors declare that the research was conducted in the absence of any commercial or financial relationships that could be construed as a potential conflict of interest.

## Footnotes

1 https://www.heliagene.org/PlasmoparaSpecies

2 http://psort.hgc.jp/form.html

3 https://www.heliagene.org/

## References

[B1] As-sadi F. Carrere S. Gascuel Q. Hourlier T. Rengel D. Le Paslier M.-C. (2011). Transcriptomic analysis of the interaction between *Helianthus annuus* and its obligate parasite *Plasmopara halstedii* shows single nucleotide polymorphisms in CRN sequences. *BMC Genomics* 12:498 10.1186/1471-2164-12-498 PMC3204308 21988821

[B2] Bos J. I. B. Kanneganti T.-D. Young C. Cakir C. Huitema E. Win J. (2006). The C-terminal half of *Phytophthora infestans* RXLR effector AVR3a is sufficient to trigger R3a-mediated hypersensitivity and suppress INF1-induced cell death in *Nicotiana benthamiana*. *Plant J.* 48 165–176. 10.1111/j.1365-313X.2006.02866.x 16965554

[B3] Bouzidi M. F. Badaoui S. Cambon F. Vear F. Tourvieille de Labrouhe D. Nicolas P. (2002). Molecular analysis of a major locus for resistance to downy mildew in sunflower with specific PCR-based markers. *Theor. Appl. Genet.* 104 592–600. 10.1007/s00122-001-0790-3 12582663

[B4] Bozkurt T. O. Schornack S. Banfield M. J. Kamoun S. (2012). Oomycetes, effectors, and all that jazz. *Curr. Opin. Plant Biol.* 15 483–492. 10.1016/j.pbi.2012.03.008 22483402

[B5] Bozkurt T. O. Schornack S. Win J. Shindo T. Ilyas M. Oliva R. (2011). *Phytophthora infestans* effector AVRblb2 prevents secretion of a plant immune protease at the haustorial interface. *Proc. Natl. Acad. Sci. U.S.A.* 108 20832–20837. 10.1073/pnas.1112708109 22143776 PMC3251060

[B6] Burrus M. Molinier J. Himber C. Hunold R. Bronner R. Rousselin P. (1996). *Agrobacterium*-mediated transformation of sunflower (*Helianthus annuus* L). shoot apices: transformation patterns. *Mol. Breed.* 2 329–338. 10.1007/BF00437911

[B7] Cabral A. Oome S. Sander N. Kufner I. Nurnberger T. Van den Ackerveken G. (2012). Nontoxic Nep1-Like proteins of the downy mildew pathogen *Hyaloperonospora arabidopsidis*: repression of necrosis-Inducing activity by a surface-exposed region. *Mol. Plant Microbe Interact.* 25 697–708. 10.1094/MPMI-10-11-0269 22235872

[B8] Caillaud M. C. Piquerez S. J. M. Fabro G. Steinbrenner J. Ishaque N. Beynon J. (2012). Subcellular localization of the Hpa RxLR effector repertoire identifies a tonoplast-associated protein HaRxL17 that confers enhanced plant susceptibility. *Plant J.* 69 252–265. 10.1111/j.1365-313X.2011.04787.x 21914011

[B9] Engler C. Gruetzner R. Kandzia R. Marillonnet S. (2009). Golden gate shuﬄing: a one-pot DNA shuﬄing method based on type IIs restriction enzymes. *PLoS ONE* 4:e5553 10.1371/journal.pone.0005553 PMC2677662 19436741

[B10] Engler C. Kandzia R. Marillonnet S. (2008). A one pot, one step, precision cloning method with high throughput capability. *PLoS ONE* 3:e3647 10.1371/journal.pone.0003647 PMC2574415 18985154

[B11] Fawke S. Doumane M. Schornack S. (2015). Oomycete interactions with plants: infection strategies and resistance principles. *Microb. Mol. Biol. Rev.* 79 263–280. 10.1128/MMBR.00010-15 PMC4468149 26041933

[B12] Fliegmann J. Jauneau A. Pichereaux C. Rosenberg C. Gasciolli V. Timmers A. C. (2016). LYR3, a high-affinity LCO-binding protein of *Medicago truncatula*, interacts with LYK3, a key symbiotic receptor. *FEBS Lett.* 590 1477–1487. 10.1002/1873-3468.12191 27129432

[B13] Franchel J. Bouzidi M. F. Bronner G. Vear F. Nicolas P. Mouzeyar S. (2013). Positional cloning of a candidate gene for resistance to the sunflower downy mildew, *Plasmopara halstedii* race 300. *Theor. Appl. Genet.* 126 359–367. 10.1007/s00122-012-1984-6 23052021

[B14] Froidure S. Canonne J. Daniel X. Jauneau A. Briere C. Roby D. (2010). AtsPLA(2)-alpha nuclear relocalization by the *Arabidopsis* transcription factor AtMYB30 leads to repression of the plant defense response. *Proc. Natl. Acad. Sci. U.S.A.* 107 15281–15286. 10.1073/pnas.1009056107 20696912 PMC2930548

[B15] Gascuel Q. Bordat A. Sallet E. Pouilly N. Carrere S. Roux F. (2016). Effector polymorphisms of the sunflower downy mildew pathogen *Plasmopara halstedii* and their use to identify pathotypes from field isolates. *PLoS ONE* 11:e0148513 10.1371/journal.pone.0148513 PMC4742249 26845339

[B16] Gascuel Q. Martinez Y. Boniface M. C. Vear F. Pichon M. Godiard L. (2015). The sunflower downy mildew pathogen *Plasmopara halstedii*. *Mol. Plant Pathol.* 16 109–122. 10.1111/mpp.12164 25476405 PMC6638465

[B17] Gessler C. Pertot I. Perazzolli M. (2011). *Plasmopara viticola*: a review of knowledge on downy mildew of grapevine and effective disease management. *Phytopathol. Mediterr.* 50 3–44.

[B18] Grant M. R. Godiard L. Straube E. Ashfield T. Lewald J. Sattler A. (1995). Structure of the *Arabidopsis* RPM1 gene enabling dual-specificity disease resistance. *Science* 269 843–846. 10.1126/science.7638602 7638602

[B19] Grant S. R. Fisher E. J. Chang J. H. Mole B. M. Dangl J. L. (2006). Subterfuge and manipulation: type III effector proteins of phytopathogenic bacteria. *Annu. Rev. Microbiol.* 60 425–449. 10.1146/annurev.micro.60.080805.142251 16753033

[B20] Haas B. J. Kamoun S. Zody M. C. Jiang R. H. Y. Handsaker R. E. Cano L. M. (2009). Genome sequence and analysis of the Irish potato famine pathogen *Phytophthora infestans*. *Nature* 461 393–398. 10.1038/nature08358 19741609

[B21] Hein I. Birch P. R. J. Danan S. Lefebvre V. Odeny D. A. Gebhardt C. (2009). Progress in mapping and cloning qualitative and quantitative resistance against *Phytophthora infestans* in potato and its wild relatives. *Potato Res.* 52 215–227. 10.1007/s11540-009-9129-2

[B22] Jo Y. Cho W. K. Rim Y. Moon J. Chen X. Y. Chu H. (2011). Plasmodesmal receptor-like kinases identified through analysis of rice cell wall extracted proteins. *Protoplasma* 248 191–203. 10.1007/s00709-010-0251-4 21161304 PMC3111878

[B23] Jones J. D. G. Dangl J. L. (2006). The plant immune system. *Nature* 444 323–329. 10.1038/nature05286 17108957

[B24] Jung S. K. Lindenmuth B. E. McDonald K. A. Hwang M. S. Bui M. Q. N. Falk B. W. (2014). *Agrobacterium tumefaciens* mediated transient expression of plant cell wall-degrading enzymes in detached sunflower leaves. *Biotechnol. Prog.* 30 905–915. 10.1002/btpr.1888 25180328

[B25] Knittel N. Gruber V. Hahne G. Lenee P. (1994). Transformation of sunflower (*Helianthus annuus* L).- a reliable protocol. *Plant Cell Rep.* 14 81–86. 10.1007/BF00233766 24192870

[B26] Krenek P. Samajova O. Luptovciak I. Doskocilova A. Kornis G. Samaj J. (2015). Transient plant transformation mediated by *Agrobacterium tumefaciens*: principles, methods and applications. *Biotechnol. Adv.* 33 1024–1042. 10.1016/j.biotechadv.2015.03.012 25819757

[B27] Lamond A. I. Spector D. L. (2003). Nuclear speckles: a model for nuclear organelles. *Nat. Rev. Mol. Cell Biol.* 4 605–612. 10.1038/nrm1172 12923522

[B28] Li Q. Zhang M. X. Shen D. Y. Liu T. L. Chen Y. Y. Zhou J. M. (2016). A *Phytophthora sojae* effector PsCRN63 forms homo-/heterodimers to suppress plant immunity via an inverted association manner. *Sci. Rep.* 6:26951 10.1038/srep26951 PMC4886637 27243217

[B29] Liu T. Ye W. Ru Y. Yang X. Gu B. Tao K. (2011). Two host cytoplasmic effectors are required for pathogenesis of *Phytophthora sojae* by suppression of host defenses. *Plant Physiol.* 155 490–501. 10.1104/pp.110.166470 21071601 PMC3075790

[B30] Lucas O. Kallerhoff J. Alibert G. (2000). Production of stable transgenic sunflowers (*Helianthus annuus* L.). from wounded immature embryos by particle bombardment and co-cultivation with *Agrobacterium tumefaciens*. *Mol. Breed.* 6 479–487. 10.1023/A:1026583931327

[B31] Malone-Schoneberg J. Scelonge C. J. Burrus M. Bidney D. L. (1994). Stable transformation of sunflower using *Agrobacterium* and split embryonic axis explants. *Plant Sci.* 103 199–207. 10.1016/0168-9452(94)90208-9

[B32] Manavella P. A. Chan R. L. (2009). Transient transformation of sunflower leaf discs via an *Agrobacterium*-mediated method: applications for gene expression and silencing studies. *Nat. Protoc.* 4 1699–1707. 10.1038/nprot.2009.17 19876029

[B33] Mestre P. Carrere S. Gouzy J. Piron M. C. de Labrouhe D. T. Vincourt P. (2016). Comparative analysis of expressed CRN and RXLR effectors from two *Plasmopara* species causing grapevine and sunflower downy mildew. *Plant Pathol.* 65 767–781. 10.1111/ppa.12469

[B34] Mestre P. Piron M. C. Merdinoglu D. (2012). Identification of effector genes from the phytopathogenic Oomycete *Plasmopara viticola* through the analysis of gene expression in germinated zoospores. *Fungal Biol.* 116 825–835. 10.1016/j.funbio.2012.04.016 22749169

[B35] Michelmore R. W. Wong J. (2008). Classical and molecular genetics of *Bremia lactucae*, cause of lettuce downy mildew. *Eur. J. Plant Pathol.* 122 19–30. 10.1007/s10658-008-9305-2

[B36] Miller J. F. Gulya T. J. (1991). Inheritance of resistance to race 4 of downy mildew derived from interspecific crosses in sunflower. *Crop Sci.* 31 40–43. 10.2135/cropsci1991.0011183X003100010009x

[B37] Mouzeyar S. Tourvieille de Labrouhe D. Vear F. (1993). Histopathological studies of resistance of sunflower (*Helianthus annuus* L.). to downy mildew (*Plasmopara halstedii*). *J. Phytopathol.* 139 289–297. 10.1111/j.1439-0434.1993.tb01430.x

[B38] Pontes O. Pikaard C. S. (2008). siRNA and miRNA processing: new functions for Cajal bodies. *Curr. Opin. Genet. Dev.* 18 197–203. 10.1016/j.gde.2008.01.008 18337083 PMC2483300

[B39] Qi L. L. Foley M. E. Cai X. W. Gulya T. J. (2016). Genetics and mapping of a novel downy mildew resistance gene, Pl18, introgressed from wild *Helianthus argophyllus* into cultivated sunflower (*Helianthus annuus* L.). *Theor. Appl. Genet.* 129 741–752. 26747047 10.1007/s00122-015-2662-2

[B40] Qi L. L. Long Y. M. Jan C. C. Ma G. J. Gulya T. J. (2015). Pl17 is a novel gene independent of known downy mildew resistance genes in the cultivated sunflower (*Helianthus annuus* L.). *Theor. Appl. Genet.* 128 757–767. 10.1007/s00122-015-2470-8 25673143

[B41] Radwan O. Bouzidi M. F. Nicolas P. Mouzeyar S. (2004). Development of PCR markers for the Pl5/Pl8 locus for resistance to *Plasmopara halstedii* in sunflower, *Helianthus annuus* L. from complete CC-NBS-LRR sequences. *Theor. Appl. Genet.* 109 176–185. 15007505 10.1007/s00122-004-1613-0

[B42] Radwan O. Bouzidi M. F. Vear F. Philippon J. de Labrouhe D. T. Nicolas P. (2003). Identification of non-TIR-NBS-LRR markers linked to the Pl5/Pl8 locus for resistance to downy mildew in sunflower. *Theor. Appl. Genet.* 106 1438–1446. 10.1007/s00122-003-1196-1 12750787

[B43] Radwan O. Gandhi S. Heesacker A. Whitaker B. Taylor C. Plocik A. (2008). Genetic diversity and genomic distribution of homologs encoding NBS-LRR disease resistance proteins in sunflower. *Mol. Gen. Genomics* 280 111–125. 10.1007/s00438-008-0346-1 18553106

[B44] Reddy A. S. N. Day I. S. Göhring J. Barta A. (2012). Localization and dynamics of nuclear speckles in plants. *Plant Physiol.* 158 67–77. 10.1104/pp.111.186700 22045923 PMC3252098

[B45] Rodor J. Letelier I. Holuigue L. Echeverria M. (2010). Nucleolar RNPs: from genes to functional snoRNAs in plants. *Biochem. Soc. Trans.* 38 672–676. 10.1042/BST0380672 20298241

[B46] Schornack S. van Damme M. Bozkurt T. O. Cano L. M. Smoker M. Thines M. (2010). Ancient class of translocated oomycete effectors targets the host nucleus. *Proc. Natl. Acad. Sci. U.S.A.* 107 17421–17426. 10.1073/pnas.1008491107 20847293 PMC2951462

[B47] Sharma R. Xia X. J. Cano L. M. Evangelisti E. Kemen E. Judelson H. (2015). Genome analyses of the sunflower pathogen *Plasmopara halstedii* provide insights into effector evolution in downy mildews and *Phytophthora*. *BMC Genomics* 16:741 10.1186/s12864-015-1904-7 PMC4594904 26438312

[B48] Shaw P. Brown J. (2012). Nucleoli: composition, function, and dynamics. *Plant Physiol.* 158 44–51. 22082506 10.1104/pp.111.188052 PMC3252080

[B49] Stam R. Howden A. J. M. Delgado-Cerezo M. Amaro T. Motion G. B. Pham J. (2013a). Characterization of cell death inducing *Phytophthora capsici* CRN effectors suggests diverse activities in the host nucleus. *Front. Plant Sci.* 4:387 10.3389/fpls.2013.00387 PMC3803116 24155749

[B50] Stam R. Jupe J. Howden A. J. M. Morris J. A. Boevink P. C. Hedley P. E. (2013b). Identification and characterisation CRN effectors in *Phytophthora capsici* shows modularity and functional diversity. *PLoS ONE* 8:e59517 10.1371/journal.pone.0059517 PMC3607596 23536880

[B51] Stassen J. H. M. den Boer E. Vergeer P. W. J. Andel A. Ellendorff U. Pelgrom K. (2013). Specific In planta recognition of two GKLR proteins of the downy mildew *Bremia lactucae* revealed in a large effector screen in lettuce. *Mol. Plant Microbe Interact.* 26 1259–1270. 10.1094/MPMI-05-13-0142-R 23883357

[B52] Stassen J. H. M. Van den Ackerveken G. (2011). How do oomycete effectors interfere with plant life? *Curr. Opin. Plant Biol.* 14 407–414. 10.1016/j.pbi.2011.05.002 21641854

[B53] Thines M. Kamoun S. (2010). Oomycete-plant coevolution: recent advances and future prospects. *Curr. Opin. Plant Biol.* 13 427–433. 10.1016/j.pbi.2010.04.001 20447858

[B54] Tian M. Win J. Savory E. Burkhardt A. Held M. Brandizzi F. (2011). 454 Genome sequencing of *Pseudoperonospora cubensis* reveals effector proteins with a QXLR translocation motif. *Mol. Plant Microbe Interact.* 24 543–553. 10.1094/MPMI-08-10-0185 21261462

[B55] Torto T. A. Li S. A. Styer A. Huitema E. Testa A. Gow N. A. R. (2003). EST mining and functional expression assays identify extracellular effector proteins from the plant pathogen *Phytophthora*. *Genome Res.* 13 1675–1685. 10.1101/gr.910003 12840044 PMC403741

[B56] Tourvieille de Labrouhe D. Walser P. Joliovot D. Roche S. Serre F. Delmotte F. (2012). “Proposal for improvement of sunflower downy mildew race nomenclature,” in *Proceedings of the 18th International Sunflower Conference*, Mar del Plata 322–327.

[B57] Trehin C. Schrempp S. Chauvet A. Berne-Dedieu A. Thierry A. M. Faure J. E. (2013). QUIRKY interacts with STRUBBELIG and PAL OF QUIRKY to regulate cell growth anisotropy during *Arabidopsis* gynoecium development. *Development* 140 4807–4817. 10.1242/dev.091868 24173806

[B58] Vear F. Gentzbittel L. Philippon J. Mouzeyar S. Mestries E. Roeckel-Drevet P. (1997). The genetics of resistance to five races of downy mildew (*Plasmopara halstedii*). in sunflower (*Helianthus annuus* L.). *Theor. Appl. Genet.* 95 584–589. 10.1007/s001220050599

[B59] Vincourt P. As-sadi F. Bordat A. Langlade N. B. Gouzy J. Pouilly N. (2012). Consensus mapping of major resistance genes and independent QTL for quantitative resistance to sunflower downy mildew. *Theor. Appl. Genet.* 125 909–920. 10.1007/s00122-012-1882-y 22576236

[B60] Vleeshouwers V. G. A. A. Oliver R. P. (2014). Effectors as tools in disease resistance breeding against biotrophic, hemibiotrophic, and necrotrophic plant pathogens. *Mol. Plant Microbe Interact.* 27 196–206. 10.1094/MPMI-10-13-0313-IA 24405032

[B61] Vleeshouwers V. G. A. A. Rietman H. Krenek P. Champouret N. Young C. Oh S.-K. (2008). Effector genomics accelerates discovery and functional profiling of potato disease resistance and *Phytophthora infestans* avirulence genes. *PLoS ONE* 3:e2875 10.1371/journal.pone.0002875 PMC2483939 18682852

[B62] Vranceanu A. V. Pîrvu N. Stoenescu F. M. (1981). New sunflower downy mildew resistance genes and their management. *Helia* 4 23–27.

[B63] Wang Q. Han C. Ferreira A. O. Yu X. Ye W. Tripathy S. (2011). Transcriptional programming and functional interactions within the *Phytophthora sojae* RXLR effector repertoire. *Plant Cell* 23 2064–2086. 10.1105/tpc.111.086082 21653195 PMC3160037

[B64] Whisson S. C. Boevink P. C. Moleleki L. Avrova A. O. Morales J. G. Gilroy E. M. (2007). A translocation signal for delivery of oomycete effector proteins into host plant cells. *Nature* 450 115–119. 10.1038/nature06203 17914356

[B65] Xiang J. Li X. L. Wu J. Yin L. Zhang Y. L. Lu J. (2016). Studying the mechanism of *Plasmopara viticola* RxLR effectors on suppressing plant immunity. *Front. Microbiol.* 7:709 10.3389/fmicb.2016.00709 PMC4870276 27242731

[B66] Yin L. Li X. L. Xiang J. Qu J. Zhang Y. L. Dry I. B. (2015). Characterization of the secretome of *Plasmopara viticola* by de novo transcriptome analysis. *Physiol. Mol. Plant Pathol.* 91 1–10. 10.1016/j.pmpp.2015.05.002

