# Supplementary material for: RXLR and CRN Effectors from the Sunflower Downy Mildew Pathogen Plasmopara halstedii Induce Hypersensitive-Like Responses in Resistant Sunflower Lines
Source: Front Plant Sci. 2016 Dec 19;7:1887. doi: 10.3389/fpls.2016.01887 (PMC5165252; doi:10.3389/fpls.2016.01887)
Supplement: Supplementary file 1 [file Table_1.docx]

**Supplementary Material:**

**RXLR and CRN effectors from the sunflower downy mildew pathogen *Plasmopara halstedii* induce hypersensitive-like responses in resistant sunflower lines**

**Quentin Gascuel†, Luis Buendia†, Yann Pecrix, Nicolas Blanchet, Stéphane Muños, Felicity Vear, Laurence Godiard***

*Correspondence:

laurence.godiard@inra.fr

**Supplementary Table 1**

**Primer sequences used for qRT-PCR and cloning experiments**

Sequences in bold, and in bold and italics were added to clone the effector by the Gateway or Goldengate method, respectively.

| effector name | forward Q-PCR primer | reverse Q-PCR primer |
| --- | --- | --- |
| PhRXLR02 | AGGCTTCTCATCTCCAACGA | GTCGCTTCTTCCACGATTTC |
| PhRXLR03 | GTATCTCCCCAATCGTGCTC | TGAGACTGCACCACTTGGAA |
| PhRXLR08 | CCTCCGACGAGAACGATAAA | GCAATTGCCCATTTCCATAC |
| PhRXLR14 | TGAAGGAAAAACAGGGCAGT | TGCAAACCAGTTGACAAAGC |
| PhRXLR31 | CAAGATGCACAAGACGAGGA | CTTGCTGTTGCGTCATGATT |
| PhCRN37 | ATTGAGACTTCCCCGAAGGT | GGGGCTGGAGACATAATCAA |
| PhRIBS3A | TGATCCATTTACACGCAAGG | GCGTTCCGAAAAGATAGCAG |
| effector name | forward cloning primer | reverse cloning primer |
| PhRXLR02 | **GGGGACAAGTTTGTACAAAAAAGCAGGCTCC**ATGGTCTCATCGTCTATCACGAAT | **GGGGACCACTTTGTACAAGAAAGCTGGGTT**CTAATACCAATACCGATGCGAATA |
| PhRXLR03 | **GGGGACAAGTTTGTACAAAAAAGCAGGCTCC**ATGGTCTCATCGTCTATCACGAAT | **GGGGACCACTTTGTACAAGAAAGCTGGGTT**CTAGTTCCAAAACTTCTTTAACG |
| PhRXLR08 | **GGGGACAAGTTTGTACAAAAAAGCAGGCTCC**ATGTCCTCCGACGAGAACGATAA | **GGGGACCACTTTGTACAAGAAAGCTGGGTT**TTAGACGACAGGATTGGAAG |
| PhRXLR14 | **GGGGACAAGTTTGTACAAAAAAGCAGGCTCC**ATGGTACCTTCCTTCGCAGAAGAG | **GGGGACCACTTTGTACAAGAAAGCTGGGTT**CTAAGGTATTTTTGCATAATCGATAC |
| PhRXLR31 | **GGGGACAAGTTTGTACAAAAAAGCAGGCTCC**ATGGCCGTTGCCTTTCGTCCCGC | **GGGGACCACTTTGTACAAGAAAGCTGGGTT**CTAATGATCATCAGCAAGAGCTC |
| PhCRN37 | ***GGTCTCGGGTGGAA***TGGTGAAGATCGCACGTGATGCG | ***GGTCTCCCGTA***TCATAACTTCAGGTTTATAACTGAAC |
| PhRIBS3A | nd | nd |
