# Supplementary material for: RXLR and CRN Effectors from the Sunflower Downy Mildew Pathogen Plasmopara halstedii Induce Hypersensitive-Like Responses in Resistant Sunflower Lines
Source: Front Plant Sci. 2016 Dec 19;7:1887. doi: 10.3389/fpls.2016.01887 (PMC5165252; doi:10.3389/fpls.2016.01887)
Supplement: Supplementary file 2 [file Data_Sheet_1.docx]

**Supplementary Material:**

**RXLR and CRN effectors from the sunflower downy mildew pathogen *Plasmopara halstedii* induce hypersensitive-like responses in resistant sunflower lines**

**Quentin Gascuel†, Luis Buendia†, Yann Pecrix, Nicolas Blanchet, Stéphane Muños, Felicity Vear, Laurence Godiard***

*Correspondence:

laurence.godiard@inra.fr

**Supplementary Data 1**

**Proteic and nucleic sequences of the *P.halstedii* cloned effectors and of PhRIBS3A**.

In the effector fusions, YFP and GFP were placed in N-terminal to the effector sequence deleted from its predicted signal peptide that is underlined. Putative translocation or conserved effector motifs are underlined in gray. _cds: coding sequence.

>PhRXLR02 len=114

MRISPVVLALAAFVIPSGAVSSSITNNDGTRSLRSSNPEPAAIADLVPALQGSDSTKRLLISNDDFDYDDHEERKSWKKR

HKKMAKYHKKYMKQYMKQYMKPWKKSKYSHRYWY

>PhRXLR03 len=100

MRISPIVLALAAFVIPSGAVSSSITNNDGTRSLRTSNPEPAAIADLMPALQGSDSTKRLLISNDDFDYDGHEERKSKKKQ

KKKAKKMAKRFKKSLKKFWN

>PhRXLR08 len=151 MRVVLLLLLTIAVSVTYVLASSDENDKALHTSDTTYGNGQLLSNEAANTETPTHDDEERNRPIGQLMSDYLFKFKMMSKT

ALWNANKIAAYLKKKNITAKKWHRVYQLQKQKNQEAKLPDGSYVKSLEQEVYELLTASQNIQKTTTSNPVV

>PhRXLR14 len=274 MCKFVLLLVIIVNGVLSVPSFAEEWESEPRVFAQRDSNTNRMLQADEHVHALYEDRRLTVSFKPEPVNLSTLGEKSPSYL

SRIANWFKNLKTTTRLAWTLFRTGFHNPDQALLMGYTPGYYISVWRKFDPTFTYLKNPDPGGDMYQKLLRFGEYYDDLQH

HQGSIKDLAEKLSNVEKQMKDMSWISRIRARIAMWKTRRLLNKGVPEEKLLEKGVSPYLYFRWLKEKQGSIPTLQDGFVN

WFATKDAMAWLKYQRLYDKKFPYPRKGIDYAKIP

>PhRXLR31 len=180 MKIIATATILGMALLHVATPTIAAVAFRPALEADETNKQQQDAQDEERALAAENHDATASGDWNALLADRETRALAYILE

AADIIDEEDNYEEEEDDYEDEDLLASGGRRRALADGGRRRALANGGRRRALANGGRRRALANGGRRRALADGGRRRALAD

GGRRRALADGGRRRALADDH

>PhCRN37 len=418 MEMKLECAVYGEKSVFPVKITRDAKVSALQEAIFDKKRYKERYSFDGSDLTLYLAKKDDAWLNHDYSVEDVLQRKIDSGY

KKMLSSRKLDDDEYFKKKFQPGDKEIHVLVELPEAAAGEKLQVVQIKRKRYVHSEMSSNEGKALLHDLNIRVKPVGTVAF

TARNPAPVQGFKWDSVCDGRGQNIALTEEQQRERYREYVEHNIGDVLAEKKLCVLGVEKGNNILSVAVPSHDIDLVGKMD

ILVLSALAKQFPHYVELLPDVKMLIEVKKVVKAGSSFQALSELIALDFLVDDPVMALLTNLTDHWQFFWVSEKNNNYVII

QTTTVTEPGAAFAVIRTLLAHSPTDDADITLPCFEKPIKRRKLAKVLPTISEVGESSGIRAAIERYYDIASVLGPDIDMA

RAAANQIARTIPVFSYYT

>PhRIBS3A len=261 MAVGKNKRLTKGKKGGKKKVVDPFTRKDWYDIKAPAIFSERNCGKTLVNRTAGTKIASEGLRGRVFEVCLADLNKDEDQA

FRKIRLCAEEIQGNQIITGFHGMDFTRDKLCSLIRKWQTLIEAFVDVKTTDGYLVRLFCIAFTKKRPNQIKKTTYAKTAQ

IRAIRKKMTSIMTDEASKCDIKDLFLKFVPEIIGKEIEKATQGIYPLQNVYIRKCKILKKPKFDLVRLMELHEGGAEEKG

AKVVRQEDQLVESMAGSGGRL

>PhRXLR02_cds len=345 ATGCGTATCTCCCCCGTCGTGCTCGCGTTGGCAGCTTTCGTGATTCCAAGTGGTGCAGTCTCATCGTCTATCACGAATAA

CGATGGTACAAGATCTCTGCGTTCAAGTAACCCTGAGCCAGCCGCTATAGCAGACCTGGTGCCCGCGTTGCAAGGCTCTG

ACAGTACAAAGAGGCTTCTCATCTCCAACGATGATTTTGATTACGATGATCATGAAGAAAGGAAATCGTGGAAGAAGCGA

CATAAAAAGATGGCGAAGTATCACAAAAAGTATATGAAGCAATACATGAAGCAATATATGAAGCCCTGGAAAAAGAGTAA

ATATTCGCATCGGTATTGGTATTGA

>PhRXLR03_cds len=303 ATGCGTATCTCCCCAATCGTGCTCGCGTTGGCAGCCTTCGTGATTCCAAGTGGTGCAGTCTCATCGTCTATCACGAATAA

CGATGGTACAAGATCTCTGCGTACAAGTAACCCTGAGCCAGCTGCTATAGCAGACCTGATGCCCGCATTGCAAGGCTCCG

ACAGTACTAAGAGGCTTCTCATCTCCAACGATGATTTTGATTACGATGGTCATGAAGAGAGGAAATCGAAGAAGAAGCAA

AAAAAAAAAGCTAAAAAGATGGCGAAGCGTTTTAAAAAGTCGTTAAAGAAGTTTTGGAACTAG

>PhRXLR08_cds len=456

ATGCGTGTGGTTTTGCTTCTACTCCTGACTATTGCCGTCTCAGTCACCTATGTCTTGGCATCCTCCGACGAGAACGATAA

AGCTCTACACACATCCGATACAACGTATGGAAATGGGCAATTGCTGAGCAACGAGGCAGCTAACACTGAGACCCCAACTC

ATGATGATGAAGAGAGAAACCGTCCGATCGGGCAGCTGATGAGCGATTACTTGTTTAAATTCAAGATGATGTCGAAAACA

GCTCTCTGGAATGCTAACAAAATTGCTGCATACTTAAAGAAGAAAAATATTACAGCGAAAAAGTGGCACCGAGTTTACCA

GCTCCAGAAACAAAAAAACCAAGAAGCAAAGTTACCTGATGGAAGTTATGTCAAATCGCTCGAGCAAGAAGTTTATGAAT

TACTTACTGCCTCTCAGAACATACAAAAAACGACTACTTCCAATCCTGTCGTCTAA

>PhRXLR14_cds len=825

ATGTGCAAGTTTGTTCTTCTCCTCGTGATTATCGTCAACGGCGTCCTCTCGGTACCTTCCTTCGCAGAAGAGTGGGAGTCCGAGCCAAGGGTTTTTGCGCAGAGAGACAGCAATACAAATCGTATGCTCCAAGCAGACGAACACGTTCATGCTTTATACGAAGATCGAAGGCTGACGGTTAGTTTTAAACCCGAGCCAGTAAATTTGTCGACATTGGGGGAAAAATCACCTAGTTATTTGTCTCGAATTGCGAATTGGTTTAAAAACCTGAAAACGACTACCAGATTAGCATGGACATTATTCAGAACTGGTTTTCACAATCCAGATCAGGCACTTTTGATGGGATATACTCCTGGATACTATATCTCCGTCTGGCGCAAGTTTGATCCTACATTCACGTACCTGAAAAACCCCGACCCTGGCGGTGATATGTATCAAAAATTATTACGTTTTGGCGAATACTACGATGATCTTCAACATCATCAAGGCTCAATCAAAGACTTAGCTGAAAAACTGAGTAACGTGGAAAAACAAATGAAAGATATGTCTTGGATCAGTCGCATTCGCGCTCGCATTGCAATGTGGAAAACAAGGCGCCTCCTCAATAAAGGTGTTCCTGAAGAAAAGTTGCTCGAGAAAGGCGTTTCGCCTTACTTGTACTTTAGGTGGCTGAAGGAAAAACAGGGCAGTATACCTACGCTTCAAGACGGCTTTGTCAACTGGTTTGCAACTAAAGACGCCATGGCATGGTTGAAATATCAGCGTTTGTATGATAAGAAGTTTCCTTATCCGAGAAAGGGTATCGATTATGCAAAAATACCTTAA

>PhRXLR31_cds len=543

ATGAAGATCATTGCGACAGCAACAATTCTTGGCATGGCATTACTCCATGTTGCAACCCCAACGATTGCTGCCGTTGCCTTTCGTCCCGCACTCGAAGCAGACGAAACGAATAAGCAGCAACAAGATGCACAAGACGAGGAACGAGCACTTGCTGCTGAGAATCATGACGCAACAGCAAGTGGAGACTGGAATGCTCTTCTTGCGGATCGAGAGACGCGTGCTCTAGCGTATATTCTTGAGGCAGCAGATATTATCGATGAAGAAGATAACTATGAGGAAGAGGAGGACGACTATGAAGATGAAGATTTGCTTGCATCTGGTGGAAGACGCCGTGCACTAGCTGATGGTGGACGACGTCGAGCGCTAGCGAATGGTGGAAGACGTCGAGCGCTTGCTAATGGTGGAAGACGTCGAGCGCTTGCTAATGGTGGAAGACGTCGAGCTCTGGCTGACGGTGGAAGACGTCGTGCTCTTGCTGATGGTGGAAGACGTCGAGCTCTTGCTGATGGTGGAAGACGTCGAGCTCTTGCTGATGATCATTAG

>PhCRN37_cds len=1257 ATGGAGATGAAGCTGGAGTGCGCAGTGTATGGCGAAAAGAGCGTGTTTCCCGTGAAGATCACTCGTGATGCTAAAGTGAG

CGCATTGCAGGAGGCCATCTTCGACAAGAAGCGATATAAAGAGCGCTACAGCTTTGATGGAAGCGATTTGACGCTGTACT

TGGCGAAGAAGGACGACGCGTGGTTAAATCATGATTACAGTGTGGAGGACGTGCTGCAAAGAAAGATCGACAGCGGATAC

AAGAAGATGCTCTCGTCGCGGAAACTCGACGATGATGAGTATTTCAAAAAGAAGTTTCAGCCTGGAGATAAAGAGATTCA

CGTCTTGGTGGAACTGCCAGAAGCTGCCGCTGGTGAGAAATTGCAAGTTGTACAAATCAAGCGTAAGCGATACGTCCATT

CGGAAATGAGCTCGAACGAGGGAAAAGCGCTGCTGCATGACCTGAATATTCGAGTGAAACCTGTTGGCACGGTTGCATTT

ACTGCTAGAAACCCAGCTCCGGTTCAGGGATTCAAGTGGGATAGCGTTTGCGATGGACGTGGTCAGAACATTGCGCTCAC

CGAGGAGCAGCAACGAGAGCGATACCGCGAGTACGTGGAACACAACATTGGCGATGTGCTCGCGGAGAAGAAGTTATGCG

TGCTCGGCGTGGAAAAAGGCAATAACATTCTCAGCGTTGCAGTACCTAGTCACGATATTGATCTTGTTGGAAAGATGGAT

ATCCTCGTATTGAGTGCTCTCGCAAAACAATTCCCGCATTATGTGGAGCTGTTGCCTGATGTGAAGATGCTGATCGAAGT

TAAGAAGGTGGTAAAAGCAGGTTCCAGCTTCCAGGCTTTGTCGGAACTAATCGCACTGGATTTCCTCGTCGATGACCCAG

TGATGGCACTGCTAACTAACTTGACTGATCATTGGCAGTTCTTCTGGGTTTCCGAGAAGAATAACAATTATGTCATCATC

CAGACAACGACCGTGACCGAACCAGGCGCAGCTTTTGCCGTGATCAGGACGCTACTTGCGCATTCACCGACTGATGATGC

AGATATCACTCTTCCATGCTTTGAAAAACCCATCAAGCGACGCAAGCTGGCGAAGGTGTTGCCGACAATCAGCGAAGTGG

GTGAAAGTAGCGGCATACGTGCCGCTATTGAACGATACTATGATATCGCAAGCGTGCTGGGTCCTGATATTGACATGGCG

CGTGCTGCGGCTAACCAAATCGCTCGAACTATTCCCGTTTTTAGTTACTACACGTGA

>PhRIBS3A_cds len=786

ATGGCTGTCGGAAAGAACAAACGTTTGACTAAAGGCAAAAAAGGCGGCAAGAAAAAAGTCGTTGATCCATTTACACGCAAGGACTGGTACGATATCAAAGCCCCTGCTATCTTTTCGGAACGCAACTGCGGTAAGACTCTGGTGAACCGTACCGCTGGTACCAAGATCGCTTCGGAGGGTCTCCGTGGCCGTGTGTTCGAGGTCTGCCTTGCGGACTTGAACAAAGATGAGGACCAGGCTTTTCGTAAAATTCGCCTTTGCGCGGAGGAGATTCAGGGCAACCAGATTATCACTGGCTTCCATGGCATGGATTTTACGCGTGACAAGCTGTGCTCGCTGATCCGCAAGTGGCAAACGTTGATTGAAGCGTTTGTGGATGTTAAGACCACGGATGGCTATCTAGTGCGTCTATTCTGCATTGCTTTTACTAAGAAGCGCCCGAATCAGATTAAAAAGACAACTTACGCTAAGACAGCTCAGATTCGTGCTATCCGTAAAAAGATGACTTCAATCATGACGGACGAAGCCTCGAAGTGCGACATTAAGGACCTTTTTTTGAAATTTGTGCCCGAGATCATTGGAAAGGAGATTGAAAAGGCTACTCAAGGCATTTACCCACTTCAAAATGTATACATCCGCAAGTGCAAGATTCTCAAGAAGCCCAAATTCGACCTGGTGCGTCTTATGGAACTCCATGAGGGCGGTGCTGAAGAGAAGGGCGCCAAGGTTGTTCGTCAGGAGGATCAACTAGTGGAATCGATGGCTGGCTCTGGTGGCCGCTTGTAG
